# Orientation of Laurdan in Phospholipid Bilayers Influences Its Fluorescence: Quantum Mechanics and Classical Molecular Dynamics Study †

**DOI:** 10.3390/molecules23071707

**Published:** 2018-07-13

**Authors:** Mirza Wasif Baig, Marek Pederzoli, Piotr Jurkiewicz, Lukasz Cwiklik, Jiri Pittner

**Affiliations:** 1 J. Heyrovský Institute of Physical Chemistry, Czech Academy of Sciences, Dolejškova 3, 18223 Prague, Czech Republic; wasifbaig.mirza@jh-inst.cas.cz (M.W.B.); marek.pederzoli@jh-inst.cas.cz (M.P.); piotr.jurkiewicz@jh-inst.cas.cz (P.J.); lukasz.cwiklik@jh-inst.cas.cz (L.C.); 2 Faculty of Science, Department of Physical and Macromolecular Chemistry, Charles University in Prague, Hlavova 8, 12840 Prague, Czech Republic; 3 Institute of Organic Chemistry and Biochemistry, Academy of Sciences of the Czech Republic, Flemingovonám 2, 16610 Prague, Czech Republic

**Keywords:** fluorescence, Laurdan, DFT, TDDFT, classical molecular dynamics

## Abstract

Fluidity of lipid membranes is known to play an important role in the functioning of living organisms. The fluorescent probe Laurdan embedded in a lipid membrane is typically used to assess the fluidity state of lipid bilayers by utilizing the sensitivity of Laurdan emission to the properties of its lipid environment. In particular, Laurdan fluorescence is sensitive to gel vs liquid–crystalline phases of lipids, which is demonstrated in different emission of the dye in these two phases. Still, the exact mechanism of the environment effects on Laurdan emission is not understood. Herein, we utilize dipalmitoylphosphatidylcholine (DPPC) and dioleoylphosphatidylcholine (DOPC) lipid bilayers, which at room temperature represent gel and liquid–crystalline phases, respectively. We simulate absorption and emission spectra of Laurdan in both DOPC and DPPC bilayers with quantum chemical and classical molecular dynamics methods. We demonstrate that Laurdan is incorporated in heterogeneous fashion in both DOPC and DPPC bilayers, and that its fluorescence depends on the details of this embedding.

## 1. Introduction

Fluorescence spectroscopy and fluorescence microscopy are key methods for studying phase behavior in biological lipid membranes [1]. Such a phase behavior determines mobility of membrane lipids and proteins being crucial for the functioning of living organisms. In this respect, fluorescence methods often rely on membrane fluorescent probes that report on their lipid environment. Laurdan is probably the most often used probe for the indication of lipid phase behavior [2,3]. In particular, the so-called generalized polarization method [4] is frequently used to assess the fluidity of lipid membranes based on the different behavior of Laurdan emission indioleoylphosphatidylcholine (DOPC) and dipalmitoylphosphatidylcholine (DPPC) bilayers. Still, the details of Laurdan emission in lipid membranes are not fully resolved. In particular, the influence of the chromophore orientation and the depth of embedding into the lipid bilayer are not understood. In contrast to the classical view, recent theoretical works suggest heterogeneous embedding of Laurdan in lipid bilayers with consequences for its spectral properties [5].

We directly address the issue of the influence of Laurdan orientation and penetration depth in DOPC and DPPC bilayers on its fluorescence. To this end we employ a combination of classical molecular dynamics (MD) and quantum chemical TD-DFT calculations. Such a multiscale approach is required to assess both the behavior of Laurdan embedded in the membrane on tens of nanosecond timescale and the emission of the excited-state dye under the influence of the membrane environment.

The manuscript is organized as follows. The methods used in both classical and quantum calculations are described and then the results are presented. First, absorption of Laurdan is analyzed in order to benchmark the computational TD-DFT methodology. Second, behavior and emission of excited state Laurdan in lipid membranes is described. All results are obtained for both DOPC (liquid-like) and DPPC (gel-like) lipid bilayers as these two systems are crucial for understanding the changes of Laurdan emission on the alteration of the state of lipid environment. 

## 2. Results and Discussion

### 2.1. Ground State Absorption Spectra–Benchmarking the TD-DFT Method

**Figure 1:**
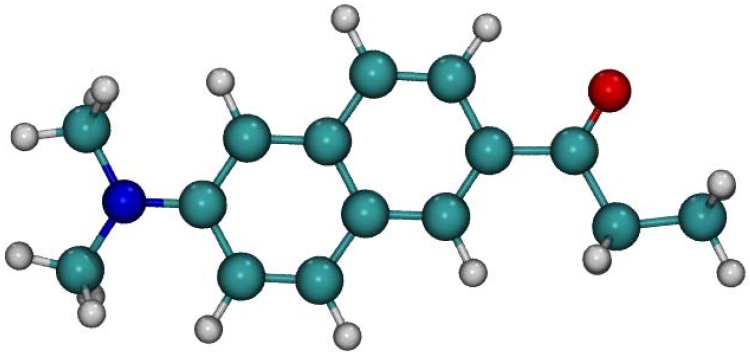
Optimized structure of ground state Laurdan fluorophore in the gas phase obtained using DFT PBE0/cc-pVDZ method. Color coding: Carbon–green, oxygen–red, nitrogen–blue, hydrogen–grey.

**Figure 2:**
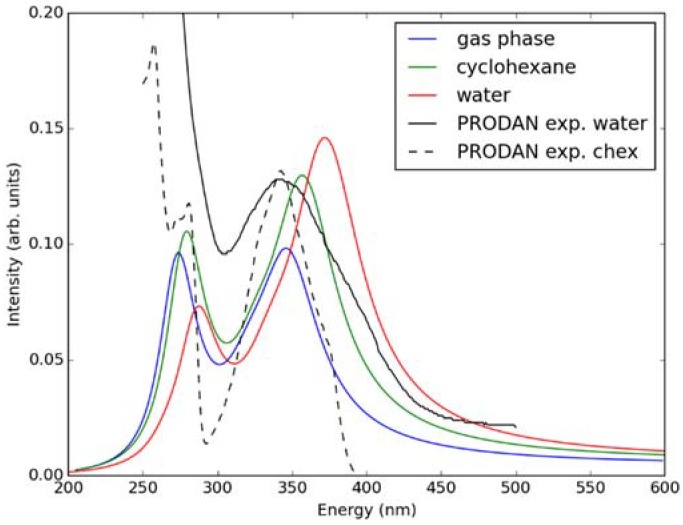
Simulated absorption spectra of LAURDAN in the gas phase, water, and cyclohexane using the TD-DFT/PBE0/cc-pVDZ method. Experimental spectra (taken from Ref. [7]) measured for PRODAN in water and experiment are shown for comparison.

Figure 1 shows the PBE0/cc-pVDZ optimized structure of Laurdan in the gas phase; the other considered methods give practically the same geometries. The molecule is planar in its ground state with the dipole moment close to 6 Debye, in agreement with earlier computational studies [6]. No significant changes of the molecular geometry employing the COSMO implicit model of solvent for water and cyclohexane with respect to the gas phase were observed. The values of the vertical absorption energies, as well as oscillator strength for Laurdan in the gas, water and cyclohexane phase computed by TD-DFT methods are presented in Table 1, Table 2 and Table 3. Overall, there is semi-quantitative agreement between all considered methods. These results are also in agreement with the values reported in earlier computational studies [6]. The magnitude of oscillatory strength demonstrates that the S_0_ to S_1_ transition is the most pronounced among the first four computed excitations while the excitation to the fifth state is beyond the energy range used in typical fluorescence experiments. Excited state energies computed from both functionals were compared with previously measured experimental data [7], and we found PBE0 to have the best agreement with the experiments. To additionally benchmark the performance of this functional for description of Laurdan excitation, the absorption spectra in the gas phase, cyclohexane, and water were calculated with five first excited states of Laurdan taken into account (see Figure 2) using the semiclassical method. These spectra are in good agreement with experimental absorption spectra measured at room temperature in water and cyclohexane for PRODAN which is the fluorescent probe having the same fluorophore as Laurdan [7]. Namely, the experimentally observed shoulder building up above 400 nm in the polar water environment with respect to nonpolar cyclohexane is reflected in the spectra calculated here as a shift of the spectrum toward higher wave numbers while going from gas phase through nonpolar cyclohexane to water. The results obtained from calculations with both cc-pVDZ and cc-pVTZ basis sets are appreciably similar. Hence, the cc-pVDZ basis set was chosen for further calculations.

### 2.2. Absorption Spectra in Lipid Bilayers

**Figure 3:**
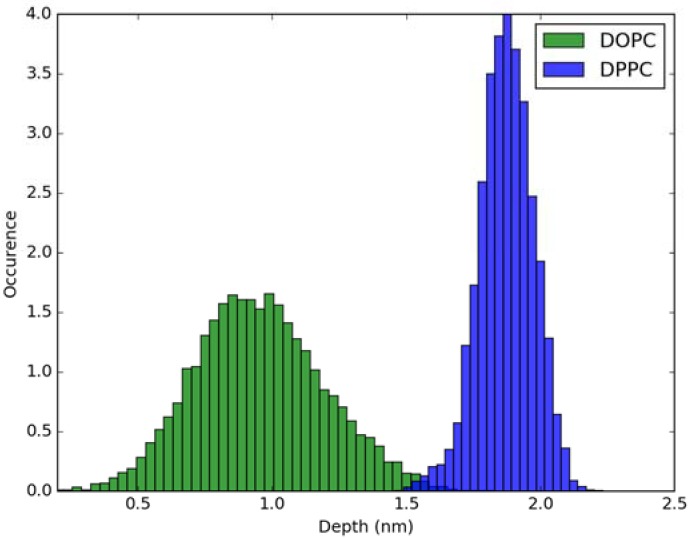
Histogram of penetration depth of Laurdan fluorophore in the ground state into lipid bilayers.

**Figure 4:**
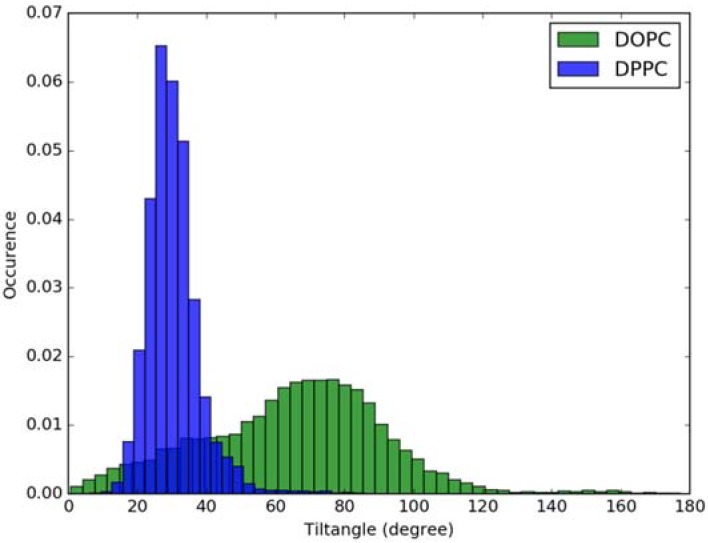
Histogram of tilt angle of Laurdan fluorophore in the ground state incorporated into lipid bilayers.

For calculation of absorption spectra, one Laurdan molecule with its complete alkyl chain incorporated in lipid bilayers was taken into account. Classical MD simulations of Laurdan in both DOPC and DPPC bilayers were performed. While the molecule of the dye stayed incorporated in the bilayers during 1000 ns-long simulations in both membranes, the details of its orientation and the depth of penetration into the lipid phase differ between DOPC and DPPC. In Figure 3 and Figure 4, histograms of fluorophore depth and tilt angle in both bilayers are shown. The tilt angle is defined as the angle between the bilayer normal and the vector connecting carbonyl the carbon atom with the nitrogen atom in Laurdan fluorophore moiety. The tilt angle value of zero corresponds to Laurdan, the fluorophore oriented in parallel to the bilayer normal with its nitrogen atom directed toward the water phase, 90 degree corresponds to the fluorophore perpendicular to the bilayer normal, and 180 degree describes a bent fluorophore oriented parallel to bilayer normal with the nitrogen atom pointing toward membrane interior. Note that in the DPPC bilayer, which under the simulated temperature is in a gel phase, lipid tails of are on average tilted with respect to bilayer normal with the most probable value of ~15 degree (as calculated here for sn-1 acyl chains of DPPC). Penetration depth is defined as the average distance along bilayer normal between the center of mass of Laurdan and the center of mass of all phosphate atoms in lipid headgroups of the bilayer leaflet with incorporated Laurdan molecule. The presented tilt angle and penetration results demonstrate that Laurdan penetrates significantly deeper into the gel phase DPPC bilayer and attains mostly orientations close to that of phospholipid tails (most probable tilt angle of ~28 degree with respect to ~15 degree for sn-1 chains of DPPC). This is in accord with the rigid nature of gel phase DPPC membranes where orientation of fluorophore “follows” that of lipids. In contrast, in the case of DOPC, penetration is not that deep and its distribution is wide. This can be rationalized by the less rigid nature of DOPC membrane which under the considered conditions is in the liquid disordered phase. Penetration of Laurdan and its accumulation between phospholipids is mostly the result of a competition between the overall attractive Laurdan-lipids tail–tail interactions and interactions (not necessarily attractive) between the fluorophore moiety of Laurdan and phospholipid head groups. The obtained results can be rationalized by prevalence of the tail–tail attractive forces in the case of well-ordered gel phase where Laurdan chain can easily attain conformation matching that of lipid tails. On the other hand, in the disordered phase it seems that for entropic reasons tail–tail interactions are weakened and hence Laurdan penetration is less pronounced. Notably, the fluorophore moiety in DOPC bilayer has more orientational freedom in that sense that it can more often attain orientations other than parallel to membrane normal. In particular, it can be oriented in perpendicular to the bilayer normal (90 degree tilt angle) as well as bend back toward membrane interior (tilt angle above 90 degree). This somewhat surprising reorientation of Laurdan chromophore was also observed in an earlier classical MD study [5].

**Figure 5:**
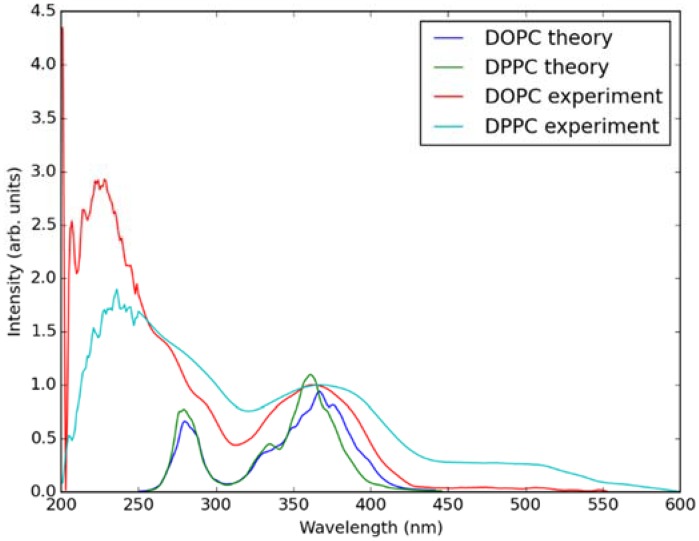
Simulated (using QM/MM MD approach) and experimentally measured absorption spectra of Laurdan in DOPC and DPPC bilayers.

In Figure 5, absorption spectra calculated using the QM/MM MD approach for Laurdan in the considered bilayers are shown together with experimental data. In the experiment, there is no significant difference between the absorption spectra measured in both lipid membranes. This is also the case in the simulated data. Note that the lower wavelength band in the calculated spectra is somewhat shifted with regard to the experiment, this is due to approximate treatment of the membrane environment. The two bands in the simulated spectra correspond to transition from π → π* and n → π* orbitals of Laurdan.

### 2.3. Emission Spectra in Lipid Bilayers

**Figure 6:**
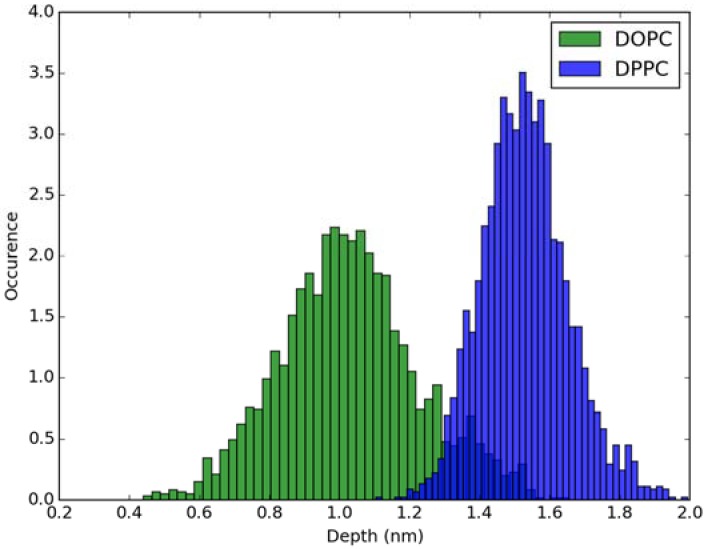
Histogram of penetration depth of Laurdan fluorophore in the S_1_ state into lipid bilayers.

**Figure 7:**
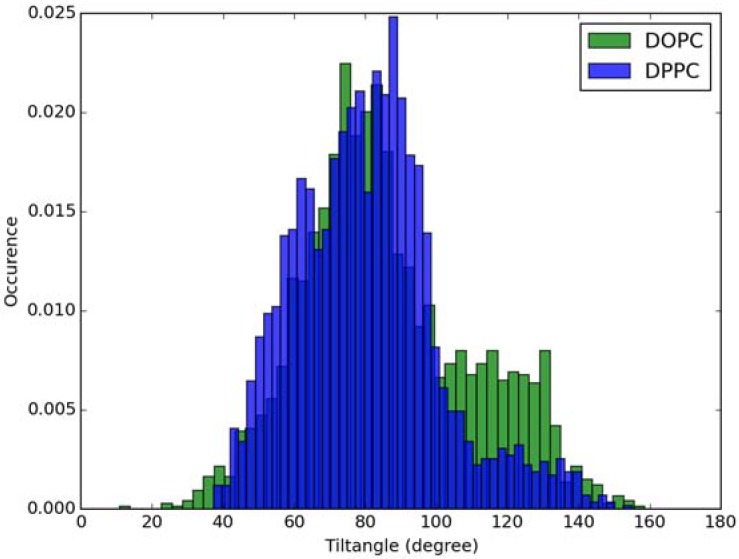
Histogram of tilt angle of Laurdan fluorophore in the S_1_ state incorporated into lipid bilayers.

**Figure 8:**
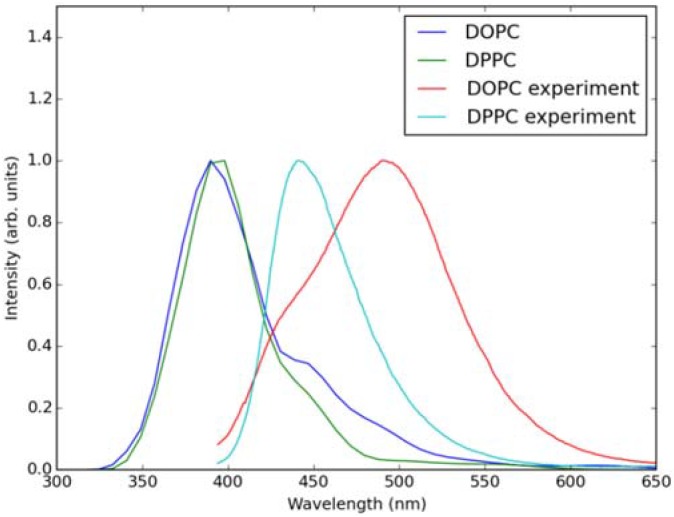
Simulated (using electrostatic embedding) and experimental emission spectra of S_1_ to S_0_ transition of Laurdan in DOPC and DPPC bilayers.

**Figure 9:**
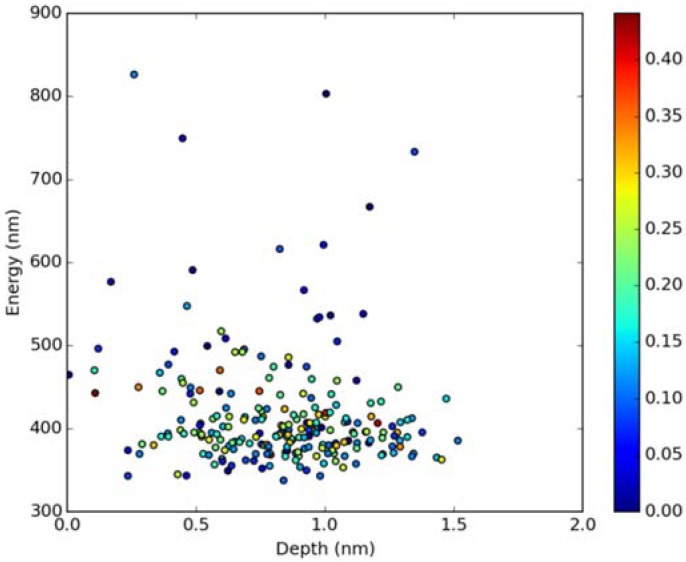
Emission energy vs. fluorophore depth in DOPC bilayer Oscillatory strength is color-coded.

**Figure 10:**
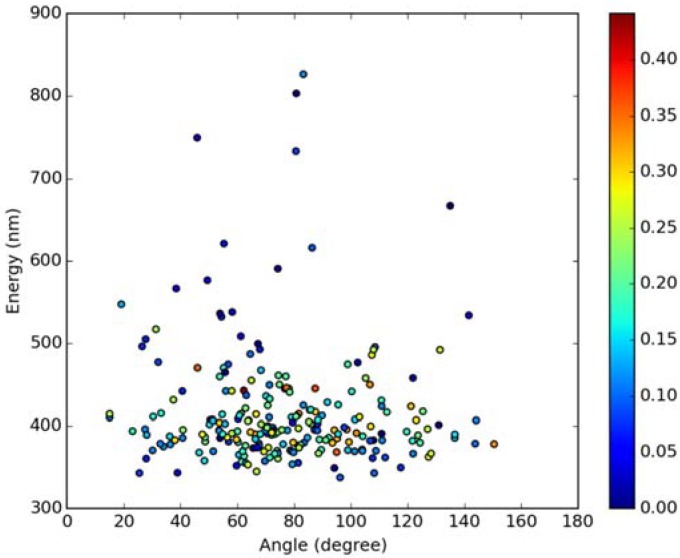
Emission energy vs. fluorophore tilt angle in DOPC bilayer. Oscillatory strength is color-coded.

**Figure 11:**
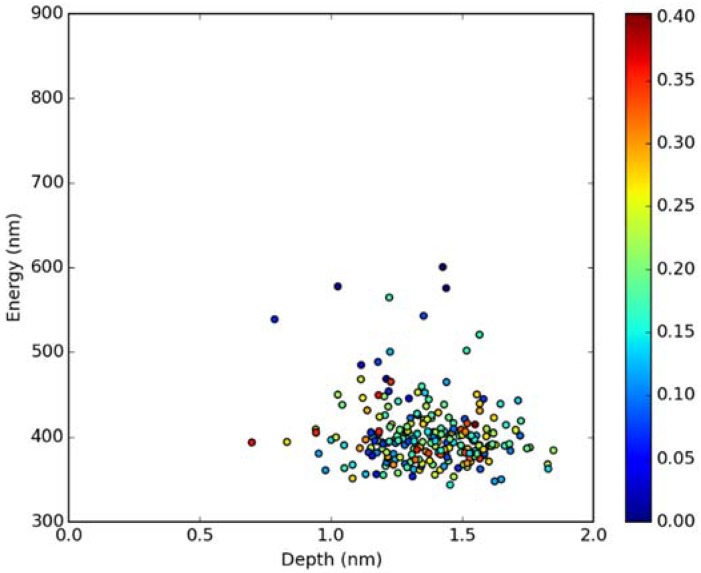
Emission energy vs. fluorophore depth in DPPC bilayer. Oscillatory strength is color-coded.

**Figure 12:**
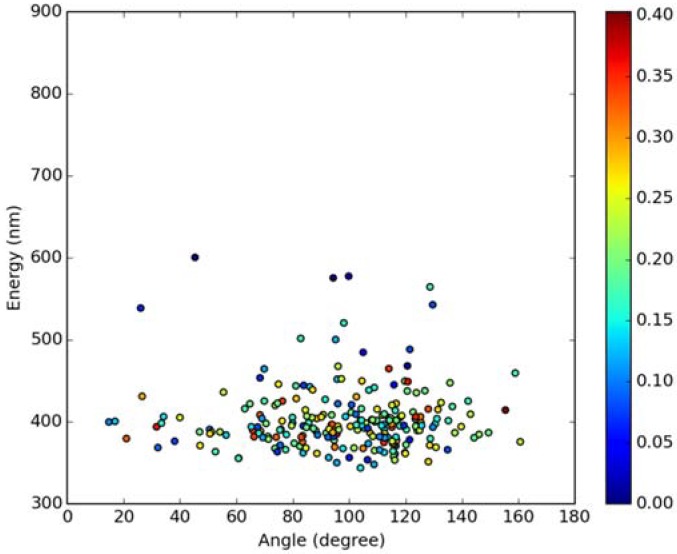
Emission energy vs. fluorophore tilt angle in DPPC bilayer. Oscillatory strength is color-coded.

Classical MD simulations of the Laurdan molecule in the S_1_ state incorporated in both DOPC and DPPC bilayers were performed. While depth of membrane penetration (Figure 6) is similar to that in the ground state, the fluorophore tilt angle (Figure 7) is significantly different. Still, penetration is deeper and orientational freedom is bigger in the case of the less rigid DOPC. Notably, the tilt angle distributions, in contrast to the ground state, are bimodal for both membranes; the higher-angle orientation is however much more pronounced in the DOPC membrane. In order to simulate emission spectra in each of the membrane, short MD polarizable trajectories were calculated starting from initial points along the nonpolarizable classical MD run. Then, an electrostatic embedding approach was used taking multiple snapshots from these polarizable calculations and calculating S_1_→S_0_ vertical transition parameters treating Laurdan at the quantum mechanical level with water and lipids described using point charges. The resulting emission spectra, together with the experimental ones, are depicted in Figure 8. There is overall no agreement between absolute positions of experimental and calculated spectra which can be rationalized by the lack of explicit water molecules it the electrostatic embedding approach. In our study, water was represented as point charges from classical trajectory and hence the results suffer from the lack of both explicit water polarizability and hydrogen bonding. Still, the calculated spectra allow us to make several qualitative conclusions when compared to experimental data. While calculated energy maxima overlap, there is a high-wavelength band visible in the spectrum simulated for DOPC. The occurrence of such a band can be directly related to the experimentally observed red shift of the emission spectra maxima between DPPC and DOPC. In order to assess the origin of the band, we analyzed in detail the dependence of the emission parameters on the fluorophore depth and orientation in lipid membranes. In Figure 9, Figure 10, Figure 11 and Figure 12, the energy dependence on depth and tilt angle of fluorophore together with oscillator strength is shown for each of 220 single point calculations in both bilayers. Even though the high-wavelength emission components in DOPC cannot be categorically assigned to specific orientation or penetration depth, the data indicate that the high-wavelength configurations occur for deep and less tilted fluorophore. This indicates that the differences in orientation of Laurdan in the more fluid DOPC and more rigid DPPC can be responsible for differences observed in the respective emission spectra.

## 3. Materials and Methods

### 3.1. Quantum Chemical Calculations

The electronic structure calculations were performed employing DFT and TD-DFT quantum chemical methods [8].Two hybrid functionals, B3-LYP and PBE0, were chosen [9,10] with cc-pVDZ, cc-pVTZ, aug-cc-pVDZ, and aug-cc-pVTZ basis sets used [11]. Since no frontier orbitals are present on the alkyl chain of Laurdan, this chain was replaced with one methyl group to decrease computational cost. Vertical excitation energies for cc-pVDZ and cc-pVTZ basis sets are quite similar and they differ significantly with aug-cc-pVDZ and aug-cc-pVTZ only for fourth and fifth excited states. Therefore, all dynamic calculations in the manuscript are done with cc-pVDZ basis set to make them computationally cheap and feasible. Geometry of Laurdan ground and excited S_1_ states in the gas phase were optimized by DFT and TD-DFT, accordingly and normal modes analysis was employed to make sure that the obtained molecular geometries are local minima. For these two structures, absorption spectra in the gas phase were computed by means of TD-DFT method using PBE0 functional with cc-pVDZ basis set. Additionally, the COSMO continuum model of solvent was employed to model Laurdan in water and cyclohexane environments. In the continuum solvent approach, the ground state optimal structure optimized in the gas phase was used. All electronic structure calculations were performed using TURBOMOLE code [12].

### 3.2. Classical MD Simulations

Classical MD simulations were performed for a single Laurdan molecule incorporated in the bilayers of both DOPC and DPPC lipids. The bilayers consisted of 128 lipids (64 in each leaflet). Note that in practical biophysical applications, fluorescent dyes such as Laurdan are typically added in <1 mol% concentrations in order to minimize potential alteration of the investigated lipid bilayer. Slipids force field was used for the description of lipids together with TIP3P parameterization for water [13,14]. The empirical force field parameters for Laurdan were derived here based on the GAFF force field and employing the Antechamber code with RESP charges calculated in Gaussian software [15,16,17]. Overall, the force field development strategy used here for Laurdan was similar to that used for the original Slipids parameterization. The S_1_ excited state force field parameters were obtained by using the ground state geometry of Laurdan with modified atomic charges. These new RESP-based charges were derived for S_1_ state of Laurdan in the TD-DFT framework. Note that since the geometries of S_0_ and S_1_ state in Laurdan are virtually the same, the modified charge distribution using ground state geometry parameters is a reasonable approximation of force field of Laurdan in the S_1_ excited state. The same approach regarding the excited state was previously used for a fluorescent dye with the same fluorophore as Laurdan [7,18]. In the case of S_1_ state, water polarizability effects were additionally considered by using Drude/SWM4-NPD force field for water [19]. This is because, based on our previous study of PRODAN fluorophore, the inclusion of water polarizability is important for description of fluorescence of the moieties similar to Laurdan [7,20]. MD trajectories of 1000 nanoseconds for S_0_ state of Laurdan were calculated in both DOPC and DPPC membranes with equilibration achieved within the first 50 ns and 350 ns for DOPC and DPPC respectively, as judged from stabilization of Laurdan orientation in the bilayer. For S_1_ state, multiple (twenty for each membrane) trajectories of 10 ns length were calculated, starting from equidistantly chosen snapshots taken from MD trajectory of S_0_ Laurdan in the corresponding lipid bilayer. Note that the lifetime of S_1_ state in lipid membranes is within this timescale. Typical parameters for membrane MD simulations were employed. Namely, periodic boundary conditions were employed with semi isotropic barostat with 1 bar pressure, and thermostat with the temperature of 305 K used. The time step of 2 fs was employed for S_0_ state calculation, and 1 fs for Drude model in S_1_ state. The cutoff of 1.4 nm was used for van der Waals and short electrostatic interactions. Long-range electrostatics was accounted for using the PME method [21]. Calculations were performed using GROMACS software suite [22].

### 3.3. Simulations of Absorption and Emission Spectra in Lipid Bilayers

Absorption spectra of Laurdan in lipid DOPC and DPPC bilayers were computed by performing QM/MM MD calculations based on the classical MD trajectories. More specifically, QM/MM MD trajectories were calculated starting from individual snapshots of Laurdan embedded in the membranes obtained from classical MD simulations in equilibrium. Thirty equidistant snapshots from each bilayer MD simulation were used for further QM/MM MD calculations. These snapshots were sampling different orientations and incorporation depths of the fluorophore in the considered membranes. These classical MD snapshots served as initial coordinates for sixty QM/MM MD trajectories; from each of those trajectories several hundreds of random geometries of Laurdan molecule were generated, and for each of these a single point calculation was performed to obtain absorption cross sections using semiclassical approximation [20]. The overall absorption spectrum was then simulated assuming Lorentzian line shape with the phenomenological broadening of 0.1 eV using Newton-X software (www.newton.org). The QM/MM MD calculations were realized using an in house code which was added to the Newton-X software suit [23] allowing for coupling quantum chemical calculations from TURBOMOLE with classical force field methods from GROMACS code [22].

Emission spectra of Laurdan in lipid bilayers were simulated employing the electrostatic embedding scheme with Laurdan treated by TD-DFT methods and the membrane and water environment accounted for by means of point charges. First, two nonpolarizable 150 ns-long classical MD simulations of S_1_ Laurdan in DOPC and DPPC bilayers were performed. Each simulation was started an equilibrated snapshot taken from the previous simulation of S_0_ Laurdan in the corresponding bilayer by changing the Laurdan force field from that describing S_0_ state to S_1_ state. Along the last 50 ns each of these two trajectories, 22 equidistant snapshots were generated, both for Laurdan in DOPC and DPPC. These snapshots served as initial geometries for 22 MD trajectories, each 2 ns-long, employing polarizable model of water; both for DOPC and DPPC. Finally, 10 snapshots were equidistantly generated between 1 and 2 ns of each polarizable trajectory; leading to 220 geometries of Laurdan embedded in the DOPC bilayer, and analogic 220 geometries for Laurdan in the DPPC bilayer. All these geometries were used for single point TD-DFT calculations of vertical S_1_→S_0_ transitions with Laurdan treated quantum mechanically at TD-DFT/PBE0/cc-pVDZ level, and water and lipids represented as point charges with point charges the values taken from the force field used in the classical MD trajectory. The resulting emission spectra of Laurdan in DOPC and DPPC bilayers were simulated based of vertical S_1_ to S_0_ transition energies and oscillator strengths assuming Lorentzian line shape with the phenomenological broadening of 0.1 eV using Newton-X software.

## 4. Conclusions

With all the limitations, the TD-DFT approach combined with classical MD simulations can be used for, at least, a semi-quantitative assessment of fluorescence behavior of Laurdan in the complex environment of lipid membranes. Absorption of Laurdan is not sensitive to its environment, as demonstrated in both DOPC and DPPC bilayers. This is similar to PRODAN and other similar probes, the ground state properties of which, are not influence by environment. On the other hand, emission properties of Laurdan in lipid bilayers are influenced by the fluorophore orientation and depth of membrane penetration. In contrast to a ‘classical view’, behavior of these two parameters for Laurdan is complex in both DOPC and DPPC. In particular, Laurdan fluorophore can not only attain conformations nearly parallel to bilayer normal but can also undergo significant conformational changes including almost complete reversal of the fluorescent moiety toward the membrane interior. Our results suggest that these heterogeneities can be responsible for the occurrence of the low-energy band of Laurdan emission in DOPC, which is crucial for understanding the methods based on Laurdan fluorescence. What is of particular importance, significant Laurdan orientational variability was demonstrated here in pure-lipid membranes. In more complicated lipid bilayers, in particular those containing cholesterol, embedded proteins and order-disorder lipid phase boundaries, orientation of Laurdan fluorophore may be further affected which suggests that the analysis of fluorescence experiments, e.g., generalized polarization, for such systems should be performed with care.

## Figures and Tables

**Table 1:** Vertical excitation energies in eV (oscillator strengths are given in parentheses) of Laurdan in the gas phase calculated employing various TD-DFT functionals and basis sets.

**Excited State**	**PBE0/aug-cc-pVDZ**	**PBE0/aug-cc-pVTZ**	**B3LYP/aug-cc-pVDZ**	**B3LYP/aug-cc-pVTZ**
**S_1_**	3.57 (0.28)	3.57 (0.28)	3.43 (0.28)	3.44 (0.28)
**S_2_**	3.81 (0.48 × 10^−4^)	3.83 (0.58 × 10^−4^)	3.71 (0.33 × 10^−3^)	3.73 (0.52 × 10^−1^)
**S_3_**	3.85 (0.77 × 10^−1^)	3.86 (0.72 × 10^−1^)	3.73 (0.55 × 10^−3^)	3.74 (0.56 × 10^−3^)
**S_4_**	4.47 (0.15 × 10^−1^)	4.49 (0.15 × 10^−1^)	4.22 (0.13 × 10^−1^)	4.23 (0.13 × 10^−1^)
**S_5_**	4.54 (0.3)	4.54 (0.31)	4.36 (0.25)	4.36 (0.26)
**Excited State**	**PBE0/cc-pVDZ**	**PBE0/cc-pVTZ**	**B3LYP/cc-pVDZ**	**B3LYP/cc-pVTZ**
**S_1_**	3.64 (0.26)	3.65 (0.27)	3.52 (0.26)	3.52 (0.27)
**S_2_**	3.83(0.21 × 10^−4^)	3.86 (0.41 × 10^−4^)	3.74 (0.15 × 10^−4^)	3.78 (0.35 × 10^−4^)
**S_3_**	3.95 (0.62 × 10^−1^)	3.94 (0.73 × 10^−1^)	3.82 (0.45 × 10^−1^)	3.81 (0.52 × 10^−1^)
**S_4_**	4.65 (0.36)	4.64 (0.34)	4.48 (0.31)	4.46 (0.29)
**S_5_**	5.13 (0.14 × 10^−3^)	5.15 (0.21 × 10^−3^)	4.88 (0.38 × 10^−4^)	4.91 (0.26 × 10^−4^)

**Table 2:** Vertical excitation energies in eV (oscillator strengths are given in parentheses) of Laurdan in the water environment modeled by implicit COSMO model calculated employing various TD-DFT functionals and basis sets.

**Excited State**	**PBE0aug-/cc-pVDZ**	**PBE0/aug-cc-pVTZ**	**B3LYP/aug-cc-pVDZ**	**B3LYP/aug-cc-pVTZ**
**S_1_**	3.33 (0.46)	3.34 (0.45)	3.19 (0.45)	3.21 (0.44)
**S_2_**	3.69 (0.89 × 10^−1^)	3.70 (0.89 × 10^−1^)	3.57 (0.62 × 10^−1^)	3.58 (0.65 × 10^−1^)
**S_3_**	3.96 (0.54 × 10^−4^)	3.98 (0.67 × 10^−4^)	3.88 (0.61 × 10^-4^)	3.92 (0.74 × 10^−4^)
**S_4_**	4.32 (0.21)	4.33 (0.23)	4.12 (0.18)	4.15 (0.20)
**S_5_**	4.67 (0.16 × 10^−1^)	4.67 (0.15 × 10^−1^)	4.43 (0.14 × 10^−1^)	4.44 (0.14 × 10^−1^)
**Excited State**	**PBE0/cc-pVDZ**	**PBE0/cc-pVTZ**	**B3LYP/cc-pVDZ**	**B3LYP/cc-pVTZ**
**S_1_**	3.44 (0.37)	3.44 (0.41)	3.32 (0.37)	3.31 (0.41)
**S_2_**	3.82 (0.10)	3.80 (0.10)	3.69 (0.73 × 10^−1^)	3.68 (0.74 × 10^−1^)
**S_3_**	3.98 (0.39 × 10^−4^)	4.04 (0.64 × 10^−4^)	3.90 (0.34 × 10^−4^)	3.97 (0.58 × 10^−4^)
**S_4_**	4.47 (0.29)	4.44 (0.27)	4.28 (0.24)	4.26 (0.22)
**S_5_**	5.11 (0.16 × 10^−1^)	5.09 (0.45)	4.95 (0.27)	4.95 (0.33)

**Table 3:** Vertical excitation energies in eV (oscillator strengths are given in parentheses) of Laurdan in the cyclohexane environment modeled by implicit COSMO model calculated employing various TD-DFT functionals and basis sets.

**Excited State**	**PBE0/aug-cc-pVDZ**	**PBE0aug-/cc-pVTZ**	**B3LYP/aug-cc-pVDZ**	**B3LYP/aug-cc-pVTZ**
**S_1_**	3.46 (0.39)	3.47 (0.39)	3.33 (0.39)	3.34 (0.39)
**S_2_**	3.79 (0.92 × 10^−1^)	3.80 (0.88 × 10^−1^)	3.67 (0.66 × 10^−1^)	3.67 (0.63 × 10^−1^)
**S_3_**	3.86 (0.58 × 10^−4^)	3.88 (0.63 × 10^−4^)	3.77 (0.52 × 10^−4^)	3.79 (0.59 × 10^-4^)
**S_4_**	4.44 (0.32)	4.44 (0.34)	4.26 (0.27)	4.26 (0.29)
**S_5_**	4.55 (0.19 × 10^−1^)	4.56 (0.18 × 10^−1^)	4.30 (0.16 × 10^−1^)	4.32 (0.16 × 10^−1^)
**Excited State**	**PBE0/cc-pVDZ**	**PBE0/cc-pVTZ**	**B3LYP/cc-pVDZ**	**B3LYP/cc-pVTZ**
**S_1_**	3.55 (0.34)	3.55 (0.36)	3.43 (0.34)	3.43 (0.37)
**S_2_**	3.89 (0.33 × 10^−4^)	3.89 (0.91 × 10^−1^)	3.77 (0.60 × 10^−1^)	3.77 (0.66 × 10^−1^)
**S_3_**	3.90 (0.82 × 10^−1^)	3.902 (0.70 × 10^−4^)	3.79 (0.52 × 10^−^)	3.84 (0.52 × 10^−4^)
**S_4_**	4.56 (0.40)	4.54 (0.38)	4.38 (0.34)	4.36 (0.33)
**S_5_**	5.15 (0.37)	5.13 (0.44)	4.95 (0.29 × 10^−3^)	4.98 (0.33)
